# Participatory co-design and normalisation process theory with staff and patients to implement digital ways of working into routine care: the example of electronic patient-reported outcomes in UK renal services

**DOI:** 10.1186/s12913-021-06702-y

**Published:** 2021-07-18

**Authors:** S. E. Knowles, A. Ercia, F. Caskey, M. Rees, K. Farrington, S. N. Van der Veer

**Affiliations:** 1 Centre for Reviews & Dissemination, University of York, York, UK; 2 Centre for Health Informatics, Division of Informatics, Imaging and Data Science, Manchester Academic Health Science Centre, University of Manchester, Manchester, UK; 3 Population Health Science Institute, Bristol Medical School, University of Bristol, Bristol, UK; 4 School of Social, Historical and Political Studies, University of Wolverhampton, Farrington, UK; 5 Renal Unit, East & North Hertfordshire NHS Trust, Stevenage, UK

## Abstract

**Background:**

Successful implementation of digital health systems requires contextually sensitive solutions. Working directly with system users and drawing on implementation science frameworks are both recommended. We sought to combine Normalisation Process Theory (NPT) with participatory co-design methods, to work with healthcare stakeholders to generate implementation support recommendations for a new electronic patient reported outcome measure (ePRO) in renal services. ePROs collect data on patient-reported symptom burden and illness experience overtime, requiring sustained engagement and integration into existing systems.

**Methods:**

We identified co-design methods that could be mapped to NPT constructs to generate relevant qualitative data. Patients and staff from three renal units in England participated in empathy and process mapping activities to understand ‘coherence’ (why the ePRO should be completed) and ‘cognitive participation’ (who would be involved in collecting the ePRO). Observation of routine unit activity was completed to understand ‘collective action’ (how the collection of ePRO could integrate with service routines).

**Results:**

The mapping activities and observation enabled the research team to become more aware of the key needs of both staff and patients. Working within sites enabled us to consider local resources and barriers. This produced ‘core and custom’ recommendations specifying core needs that could be met with customised local solutions. We identified two over-arching themes which need to be considered when introducing new digital systems (1) That data collection is physical (electronic systems need to fit into physical spaces and routines), and (2) That data collection is intentional (system users must be convinced of the value of collecting the data).

**Conclusions:**

We demonstrate that NPT constructs can be operationalised through participatory co-design to work with stakeholders and within settings to collaboratively produce implementation support recommendations. This enables production of contextually sensitive implementation recommendations, informed by qualitative evidence, theory, and stakeholder input. Further longitudinal evaluation is necessary to determine how successful the recommendations are in practice.

## Background

Digitisation of healthcare systems continues unabated. However, digital systems have provided notable examples of the challenges of implementation [1, 2]. Implementation refers to the degree to which new ways of working are adopted, integrated and sustained in practice. Failures of implementation can mean both wasted resource and also limit the extent to which benefits of new ways of working can be realised.

Systems for routinely collecting and using electronic patient-reported outcomes (ePROs) as part of clinical practice are an example where significant implementation challenges may arise. ePROs reflect the personal impact of illness and treatment as assessed by patients [3], such as symptom burden or the effect of a disease on someone’s quality of life. Routine ePRO collection has the potential to improve patient care and outcomes [4, 5], including detection of health problems that would otherwise go unnoticed. Reaching this potential requires repeated individual engagement with digital technology from patients and professionals, integration with existing information systems, and standardised delivery at scale. Yet, it is still largely unknown how best to achieve this [6].

Given the implementation challenges encountered, ePRO programmes and other digital health initiatives increasingly aim to develop contextually sensitive interventions [7, 8] to support implementation into practice. This approach recognises that ePRO systems are socio-technical. Consequently, approaches to support their implementation must be grounded in the context – clinical, social, and organisational - in which they are expected to operate. This requires understanding both the place where such interventions are intended to work and the people who are expected to carry out the work. A socio-technical approach therefore requires the use of methods which work with users and within settings.

Co-design with intended end users and iterative processes of in situ development are increasingly recognised as key components of intervention design [9]. In healthcare, end users can be both patients and professionals. Working with staff may ensure fidelity of the ePRO system, including collection and review processes, as staff can engage in covert resistance to what they perceive as disruptive digital collection protocols [10]. Similarly, including patients may account for notable differences in how service users anticipate digital interventions compared to staff [11], which can impact on patient engagement with ePRO collection and their expectations of how ePRO results are integrated into the service.

It is recommended that implementation research draws on theory to guide the development of strategies to support uptake and integration of new interventions into practice [12, 13]. Normalisation Process Theory is a theory of mechanisms that can support or impede the implementation of new ways of working, and has been used extensively in feasibility studies and process evaluations of implementation [14], in particular of digital health interventions [15]. However, studies using NPT have tended to focus on single stakeholder or user groups, and there has been less use of NPT in formatively guiding implementation approaches [16].

So, taken together, effective implementation of digital ways of working, including systems to routinely collect and use ePROs, requires approaches which:

1. Generate solutions which are feasible and acceptable in practice (sensitive to context);
2. Engage directly with end users, both patients and professionals (guided by multi-stakeholder input);
3. Incorporate theoretical insights into design and development (theory-informed).

Participatory co-design is a collaborative method which requires creative partnerships between researchers and the end users of their research, such as healthcare professionals and patients. It was developed specifically in the context of technology, to enable users to future forecast their experiences with a new technology in a formative design stage, to collectively propose solutions to improve use in practice [17]. Participatory co-design involves the use of typically visual or narrative methods to better understand the needs and circumstances of the end user [18] and rapid prototyping processes to critique assumptions and propose solutions [19]. It has been used with both patients and professionals to propose solutions to complex healthcare problems [20].

One criticism of co-design is that it privileges user feedback whilst potentially neglecting existing evidence and theory. Studies have demonstrated however that implementation theory can be effectively employed alongside participatory approaches such as Participatory Learning and Action [21]. The studies suggest that stakeholder input and theoretical insight combined provide a deeper understanding of implementation challenges and potential solutions[22] and may lead to more sustained implementation change [23]. Co-design activities can incorporate existing evidence, for example, by building user personas based on qualitative evidence syntheses, or by analysing co-design outputs using theoretical frameworks [20]. Whereas integration of theory and co-design activity is increasingly common at an individual level, e.g. by drawing on behavioural science theories to inform co-design of specific interventions through mapping specific behavioural constructs with intervention components [24], there have been fewer examples of using co-design techniques to map system-level implementation constructs.

In this study we, therefore, aimed to investigate whether participatory co-design methods could operationalise theoretical constructs (NPT), while working with multiple stakeholders to anticipate barriers to implementation. Specific objectives were to explore:

1. how these design methods could support elicitation and synthesis of relevant patient and professional expectations and concerns regarding implementation; and.
2. how NPT could be integrated directly with such methods, to derive theoretically informed recommendations for implementation in practice.

We addressed these questions in the context of a wider study that aimed to implement a system to routinely collect and use ePROs in renal services in England. We drew on NPT as a mid-level implementation theory with relevance to both individual and system levels, and integrated it with specific co-design techniques, from the field of human centred design [25], with both patient and professional stakeholders. NPT was selected based on previous success employing this method alongside participatory approaches. The co-design techniques were chosen as they enabled targeted qualitative data collection (recognising that this was a specific co-design phase to inform a wider project and so needed to blend theory with specific co-design techniques, in contrast to studies which employed participatory approaches throughout.) This enabled us to better anticipate and develop potential solutions to local implementation challenges for embedding the ePRO system into routine care, while also generating generalisable learning to prepare for spread of the system at a national level.

## Methods

Theoretically informed participatory co-design.

### Study context

In 2015, the UK’s National Health Service (NHS) launched the national Think Kidneys programme. The programme aimed to support people with chronic kidney disease (CKD) to manage and make decisions about their health (https://www.thinkkidneys.nhs.uk/). As part of Think Kidneys, PROs (i.e. symptom burden, health-related quality of life and patient activation level) were collected in over 3,000 people across fourteen renal units using paper questionnaires. Although this demonstrated enthusiasm in the renal community for collecting PROs, the programme also showed that paper-based collection was unlikely to be sustainable [26].

Digitising PRO collection would address many of the issues associated with paper-based collection. Therefore, the OPTimising engagement in routine collection of electronic Patient-Reported Outcomes (OPT-ePRO) project [27] aimed to develop a contextually sensitive and theory-informed strategy to support implementation of a system for routine collection and use of ePROs (i.e. symptom burden and health-related quality of life) into renal services [28]. Renal services are relatively digitally mature, with all treatment centres having an electronic patient record linked to a national data infrastructure including an online patient portal (https://renal.org/patients/patientview). This setting, therefore, provided an exemplar context with broad community buy-in and an established national technical infrastructure on one hand, and on the other a lack of knowledge on how to engage individual professionals and patients in delivering the ePRO system in clinical practice.

### Design

The OPT-ePRO project adopted an agile delivery design, with an early formative co-design stage to develop initial implementation support, followed by iterative cycles of refinement. This paper reports on the co-design elements in the formative stage of the study. The study design required assessment of perceived acceptability and feasibility, driven by pragmatic questions regarding organisation and delivery of support for the ePRO system. We mapped these questions to the higher level NPT constructs of coherence, cognitive participation, and collective action, and then selected appropriate participatory co-design methods to capture data relevant to that construct (see Table 1). Reflexive monitoring, the NPT construct referring to how users evaluate implementation in practice and use learning to make adaptations, was considered to best reflect the planned iterative development cycles, and data will be collected for this construct in future longitudinal evaluation. The full NPT framework, including sub-categories of the higher level contructs, can be explored online: http://www.normalizationprocess.org/.

Our co-design methods included interactive design prompts (process and empathy maps) and observation, chosen to achieve insight into NPT constructs (Table 1).

**Table 1:** Integrating NPT constructs and co-design methods to address evaluation questions

Evaluation Question	NPT Construct	Design Method
Why? *Why should patients and professionals engage with the ePRO system?*	Coherence *What is the meaning of the intervention to the different stakeholders?*	Empathy mapping Involves asking a stakeholder group to map out what they see, hear, and think in a particular situation or setting, and what their main ‘pains and gains’ are (what they hope will happen and what they are worried about) Aim: To better understand the end user of the ePRO system, to elicit data regarding the individual (personal) and social (organisational) context in which the ePRO system would be expected to operate.
Who? *Who needs to be involved in supporting delivery of the ePRO system?*	Cognitive participation *What roles need to be undertaken to deliver the intervention and who is able to perform them?*	Process mapping A flowchart style representation of a process in action over stages of time or across different locations, including specifying tasks and roles to deliver each element. Aim: to use process mapping as a form of prototyping, to communicate our expectations of how the ePRO system would be delivered in practice, and to enable staff and patients to imagine this process and expose assumptions or anticipate barriers to delivery.
Where & when? *What is the best time and location to deliver the ePRO system?*	Collective action *What are the existing routines and practices which the intervention must work alongside?*	Observation Rapid ethnography to enable in situ observation of routines of practice. Research team visits at each participating site, formal observations conducted by researcher (AE). Aim: to understand the spatial and temporal organisation of the site, and to observe how staff and patients interacted and how patient information was collected.
How? *How well has delivery of the ePRO system worked, and what could be done differently?*	Reflexive monitoring *How do users assess the value of the intervention and how do they make changes?*	Iterative development Cycles of development with each study site to identify problems in realising the value of ePRO system and implement modifications to improve delivery. (To be explored in future work)

### Sample

We conducted five co-design workshops with patients, caregivers and staff from our study sites in three NHS England Trusts. We completed empathy mapping and process mapping in each workshop. All workshops occurred between Jan- August 2019 and lasted approximately three hours. Three workshops were attended only by patients and caregivers (total n = 25). Two workshops were attended only by staff (total n = 13). We planned one of the workshops to be attended by both patients, caregivers and professionals but scheduling conflicts with staff made this impossible. Patient participants were recruited by members of their care team, who were asked if they knew of patients who would be interested in contributing to a renal service design activity, or via local patient representative groups (e.g., Kidney Patient Association). As a co-design activity, we did not apply a sampling framework to the invitation of patients beyond asking for them to have relevant lived experience and a willingness to contribute to service design. Staff were recruited via their unit manager.

### Materials and Data Collection

Both the empathy mapping and process mapping took place in co-design workshops. The observation took place at each site.

#### Co-design workshops and mapping activities

In each workshop, we first asked the participants to complete an ‘Empathy Map’ for a patient (in the patient workshops) or for an HCP on the ward (health professional workshops). Participants worked in small groups of 2–4 and completed the activity with pens and post-it notes on a large A3 template. Each group then presented their map to the wider group for further discussion.

Secondly, we presented a prototype process map of how the ePRO system could be delivered, reflecting stages such as registering patients to use the online portal for data entry, collecting ePRO data using a desktop or tablet, and presenting summaries of the collected ePRO information to staff. This prototype was drafted based on input from the early site visits and with feedback from the wider research team, including healthcare professionals. We invited participants to imagine each stage of the process and suggest potential barriers or identify missing steps. As before, participants worked in small groups of 2–4 using an A3 template. Each group then presented their map to the wider group for further discussion.

All workshops were facilitated by members of the research team (SK,AE) with experience of participatory research and co-design activities.

#### Observation

Rapid qualitative ethnography [29] was employed to enable observation of roles and routines in action [30]. Sites were initially visited by all three research team members (SK, SV, AE), including visiting the ward/consultation rooms to gain familiarity with each site’s context. Formal observation involved more immersed observation by AE who conducted 51 h across sites, guided by a field notes template. The template was informed by the evaluation questions and with feedback from clinical research team members and a service user researcher to guide data collection on key delivery activities occurring at the site. The topics on the template included:

- Patient time and activities: where do patients spend time while attending the unit? What are they doing?
- Patient interactions: who do patients speak to most? Who do they interact with, and when?
- Patient waiting period: how long do patients wait, and where does this happen?
- Staff presence and activities: which staff are present on the ward? What are they doing?
- Staff interactions: who do staff speak to? Do staff interact in groups or individually?
- Recording of patient data: who records patient data while they are on site? How is data collected?

The final recorded data set therefore comprised of completed maps and field notes collected during the workshop, and completed observational field notes.

### Analysis

The data analysis aimed to extract learning at two levels:

1. Site specific: key actionable learning in terms of support for implementation of the ePRO system in practice, including sensitivity to local differences in processes and in staff resources and configurations.
2. General thematic: to formulate broader conceptual themes to contribute to understanding socio-technical implementation of the system beyond the local context.

Analysis therefore happened in two stages: The first stage involved concurrent and collaborative analysis within each workshop, to engage the participants directly in identifying challenges and co-creating solutions. The second stage involved retrospective analysis of outputs of all the workshops and the observations, to identify common themes and generalisable learning to inform future efforts to implement the ePRO system at scale.

#### Stage One Analysis

Co-design involves collaborative real-time synthesis between workshop participants and researchers [31, 32], to generate understanding, question assumptions, and gather local feedback to inform site-specific recommendations. Analysis in co-design therefore happens concurrently with generation [33] in a dialogic process to agree key outputs.

In this study, outputs were consensus around main barriers to be encountered and suggestions for support that would overcome these problems in practice. The workshops were conducted prior to the observation data collection. Initial recommendations were therefore generated within the workshops, then reviewed with the research team, and finally were revisited after observation to check if the feasibility or acceptability of the proposed solutions was or was not confirmed by the observation data.

#### Stage Two Analysis

The second stage involved more formal qualitative analysis of the workshop outputs and observational data. Initial inductive content analysis was conducted by AE to consider the dataset as a whole and allow for unexpected findings prior to applying the conceptual framework (NPT). After agreement between authors that the NPT constructs did not exclude any coded data, the data from the patient workshops and the professional workshops were organised into tables according to NPT constructs. This enabled us to thematically review the data across the constructs to inform general recommendations and to consider the findings more broadly in the context of socio-technical interventions. The observation data was then added into the table to examine consistencies and look for any divergence in findings. The completed tables were reviewed by SK and AE to agree overarching themes. These were further sense-checked with MR, a qualitative researcher with lived experience of renal services.

## Results

Firstly, we present the site-specific learning and recommendations for implementation of the ePRO system in practice, organised according to the NPT constructs. During the study, we found that recommendations were synthesised from across the three methods (empathy mapping, process mapping, and observation) rather than each producing discrete recommendations relating independently to individual constructs. Secondly, we present the general thematic findings regarding implementation of the ePRO system as a socio-technical endeavour.

There were some differences in emphasis in the staff compared to patient workshops. Staff focused their user experience more on processes within clinical settings, which is understandable given this is where they professionally operate. Patients focused more on the relevance and delivery of the ePRO system at home and between appointments, again understandable as the majority of their experience occurs outside clinical interactions. These were not exclusive, however, with patients also discussing organisation within services (for example, receiving information in a waiting room) and with staff interested in how patients could be supported to complete their ePRO questionnaires from home. For this reason, results are presented together (reflecting both staff and patient views) rather than presenting the groups separately.

### Stage 1: Site-specific learning and recommendations for implementation

#### Coherence

The perceived value of the ePRO system was a crucial consideration for both patients and staff across all sites. Patients emphasised that completion of ePRO questionnaires would depend on knowing how it helped them to communicate their needs or concerns better to staff. This improvement could be through supporting changes to their care based on their symptom reporting, or more immediately achieved through reducing the time they spent in the clinic (e.g. if they were able to more quickly provide a report of their condition) or helping them to get a quicker response to their most pressing concern.

Staff were concerned about running over time and that the ePRO system could disrupt their routines and impede efficiency, but they were equally worried about missing something important that a patient might wish to tell them. Explicit value was not only important to initiate completion, but was returned to as a crucial consideration throughout the process of collecting and using ePROs. In the process mapping, both patients and staff would return to the question of value and how this was being communicated and realised at different timepoints (both during completion and afterwards as a prompt or review tool for future consultations). Coherence therefore overlapped with cognitive participation and collective action, in terms of key dimensions of value being “valuable to whom?” (cognitive participation) and “valuable when? “ (collective action). The observation data emphasised that achieving this value in practice was a key consideration as any information gained from the ePRO system would need to be made easily accessible within the demanding environment in which the staff and patients interacted.

#### Cognitive participation

The process maps helped in recognising the multiple practical points when different staff may be required and what experience and skills they need to support ePRO collection. For example, the process mapping revealed that ePRO reporting using tablets required consideration how the data collection could be integrated with workflow requirements around infection control. A key engagement or disengagement point was identified as the registration of patients onto the online patient portal, and considering who at the site was best placed to support the patient to do this, for example nurses, health care assistants, or reception staff. Notably, the suggested solution to this problem varied across sites, depending on local capacity and knowledge. These insights also overlapped with collective action in terms of understanding the organisational routines of the different staff (for example, who has time to circulate on the ward and offer support, or who is already involved with supporting registration).

The observation data went further to suggest that negotiation of the roles on the wards would be a key element of implementation. On the process mapping activity, it was most often a nurse who had been suggested to provide support. During the observation, it was more explicitly apparent that nurses already undertake a variety of tasks (e.g., take bloods, distribute medicine, respond to crises) which managers may be wary of disrupting by providing additional work. It was apparent during observation that healthcare assistants conduct a variety of supporting activities such as material inventories and preparing machines, and were considered more likely able to take on the ePRO support tasks. However, willingness to do this and willingness to give HCAs this responsibility varied across sites. Similarly, administrative staff varied across sites in their perceived capacity to support patient registration and access issues with the online patient portal. This therefore indicated the need for ongoing evaluation of this during the future iterative cycles to determine how work was allocated and received in practice.

#### Collective action

The observation pragmatically informed the recommendations through adding understanding of the routines on the ward and observing highly active times compared to quieter periods. This enabled us to consider the optimal timing for staff to invite patients for ePRO completion, without disrupting established clinic routines. This also contributed to cognitive participation in terms of observing how support and teaching happened on the ward (with learning shared in conversations between staff rather than through consulting written materials).

##### Translating the constructs into recommendations

Achieving and communicating the value of the ePRO system was therefore a consistent priority across both staff and patients and across settings. This became a core recommendation to achieve coherence (Table 2). For cognitive participation and collective action, there were several core recommendations around organising support and integrating with workflows, which were consistent in their relevance across sites, but which required site-specific solutions to be proposed to achieve in practice (Table 2).

**Table 2:** Example data mapped to NPT constructs, and recommendations

NPT Construct	Exemplar data	Recommendations
**Coherence** What is the meaning of the intervention to the different stakeholders?	**Staff Workshop**- Staff need to view it as an opportunity to better care for patients/ improve delivering care- *“this is innovative, interesting and important”; “What can we do better?”; “I aspire to any feedback given by the patients on how to improve the experience during dialysis”* **Patient Workshop-** Patients need to view it as an opportunity to improve communication with clinicians/ improve health condition - *“improvement in interaction with consultant”;* “getting response back”; “*improvement in health condition”*	**On-site emphasis on value, through materials and support**: Making data available to all staff after completion Presentations during team meetings with all unit staff to help them understand how this data will be used, who will use this data to emphasise the value. Local materials (FAQ sheets and invitation letters) produced for patients to inform them about the value of completing ePRO. Trained specific staff members to discuss with patients the value of completing ePRO regularly and how it would be used. Modifying patient materials to emphasise how completion can support conversations with HCPs.
**Cognitive participation** What roles need to be undertaken to deliver the intervention and who is able to perform them?	**Observations-** The haemodialysis and outpatient units operate with different level of staff with specific responsibilities. *Managers*- The unit manager can manage the intervention and assign tasks to specific staff. *Implementers*- Healthcare assistant workers and other support workers were could take on the collection of ePRO due to their availability.	**Local tailoring of who delivered which elements of the intervention**: Work with the unit manager to identify the most appropriate staff who can manage the intervention and deliver specific elements of the intervention. Individually discuss the role of consultants in reviewing the ePRO data
**Collective action** What are the existing routines and practices which the intervention must work alongside?	**Staff Workshop**- Staff emphasised the need to understand how to best embed the ePRO in their routine, to minimise disruption to other tasks. This included knowing how to support patients and answer patient questions about completion. “*How to complete [collect ePRO]”; “reassure patients that filling out the survey for their benefit”; “when is the best time/ day to do it [collect ePRO]”; “Explaining why you’re doing it”* **Patient workshop**- Patients are concerned about having space (physical and mental) and privacy, timing in relation to when it will be reviewed- *“Would I have the option to do this from home prior to my appointment?”; “the waiting room is non-existent. It is a very busy corridor and very disturbing to any thought process”; “privacy is a big issue in the so-called waiting area of my clinic”* **Observation-** Units in different sites practice symptom collection differently. Some clinicians are required to directly report symptoms in the patients’ electronic patent record. Patient symptoms are discussed during team/hand over meetings. The intervention must be embedded in the routine practice of providing dialysis in HD units. It must also be embedded in the rapid flow of outpatient units.	**Local tailoring of when and where the ePRO is completed**: Allowing the staff responsible for collecting ePRO to perform the task any time during their shift. Ongoing in-person training with selected staff to build their understanding of the aim and value of collecting ePRO. Providing reminders to patients to complete their ePRO ahead of the ePRO review to give them the opportunity to complete it in their preferred location.

### Stage 2: Learning and recommendations beyond the local context

Two intertwined themes emerged from the conceptual analysis, relating to understanding the collection of ePRO data within a socio-technical system.

#### Theme 1: digital data collection is physical

This theme emphasised that rather than being an intangible system quality, collection of ePRO data was very much a physical process in terms of how it was enacted on the ward. This included specifying how the data collection worked within complex spaces (for example, where in the busy and open-plan wards the tablets could be stored and accessed) and the physical consequences of this (requiring infection control procedures to clean tablets after each use, and a procedure for securely storing the tablets at the end of the day). This physicality of space was also evident for the patients, who emphasised the need for private and quiet space to complete their ePROs on the ward, which was typically noisy and with many distractions. The collection of ePRO data therefore makes demands on both time and space in the clinical and administrative setting, which needed to be accommodated. These accommodations were considered acceptable when the value of this additional work was made explicit, referring to the intentional quality of ePRO collection (see theme 2).

#### Theme 2: digital data collection is intentional

Across all staff and patients, it was emphasised that the value of ePRO collection must be explicit for engagement to happen. This was in recognition of the first theme: data collection made physical demands on both staff and patient resources which required deliberate and intentional engagement, and consequently there needed to be a clear rationale for why this was important. For both staff and patients, the temporal value of ePRO results needed to be negotiated, to identify how the results could add value to the clinical encounter at the time as well as how it provided a picture of progress which would be considered at a later date. For example, patients explored how they could use it as a consultation support tool to bring up important issues with their doctor. This also required understanding that users engage with ePRO collection with consideration of how value will be realised at different timepoints, for example within the next clinical consultation or through providing a picture of process over a longer period.

Both themes are consistent with NPT, emphasising the work that needs to be done to enact change (the physical integration and organisation of the intervention) and the importance of stakeholder buy-in to the need for the intervention and whether stakeholders perceived the impacts to be valuable for them.

## Discussion

We have demonstrated how human-centred co-design methods can be used to operationalise NPT constructs, to work directly with health services end users, both patients and professionals, to anticipate barriers to implementing an ePRO system in practice. The design enabled us to create overarching principles to guide implementation at scale, recognising that ePRO data collection is physical and intentional, but also to pragmatically consider how to realise these principles in action. This included understanding where solutions needed to be tailored to local site resources and capacity. The study therefore demonstrates how to design theory-driven, stakeholder informed, and contextually sensitive digital health solutions.

NPT provides a ‘conceptual vocabulary’ to guide implementation planning [16]. The co-design techniques we used enabled us to translate that vocabulary into interactive qualitative prompts, allowing us to capture rich data about stakeholder expectations and contextual barriers and facilitators. The integrated synthesis that happened within the workshops themselves supported rapid analysis to generate recommendations within the timeline of the evaluation, but also produced richer insights about the how and why of ePRO system implementation in practice. The methodology therefore supported both rapid pragmatic and deeper conceptual synthesis.

The modelling and prototyping work within co-design helps to focus on the anticipated practical realities of delivery. Although the modelling is hypothetical, the focus on the user experience appeared to help the participants with forecasting likely problems and proposing concrete solutions. Our findings are consistent with qualitative interview studies exploring perceptions of renal ePROs, which for example identified the need to support patients to complete [34] and the need for benefits of completion to be clear [35]. The collaborative co-design approach enabled us to actively recruit the local services themselves to help translate these needs into actionable changes.

The observation data expanded on the co-design suggestions, enabling us to be more sensitive to place and configurations of staff activity and patient need. Our findings suggest that observation is a crucial element as it can provide explicit confirmation of barriers hypothesised in the co-design workshops (for example, demonstrating the multitude of clinically significant tasks that nurses undertake on the ward) and also bring to light issues which were not raised by participants (the need to consider whether alternative staff, such as HCAs, were better placed to provide support, and the social dynamics between staff that may influence integration of the intervention). Our findings therefore indicate the value of combining qualitative and conceptual mapping work with direct observation. The co-design methods enable the ‘technological imagination’ of the stakeholders while the observation, by comparison, enables researchers to access the ‘technological reality’ of the context of delivery.

This also indicates the value of employing the NPT framework in conjunction with the choice of design methods, as the construct of ‘collective action’ indicated the need for observational data to supplement the mapping work. However, we did not find that the mapping techniques produced individual findings specific to individual constructs (for example, empathy mapping only indicating recommendations relevant to coherence), as in practice insights from each activity were used to reflect across the three constructs of coherence, cognitive participation, and collective action. Rather than suggesting a lack of differentiation, we suggest that this reflects the holistic understanding that was gained across the methods, with the constructs providing a valuable organising structure to develop recommendations.

### Implications

Our study adds to the literature demonstrating the need to understand routine of care into which a new digital intervention is being introduced [36], and concurs with previous authors who recommended understanding interactions with digital health interventions using observation [37] and co-design methods [19]. We have demonstrated how these methodologies can be blended with implementation theory, consistent with calls for greater use of implementation frameworks to understand engagement with both digital health technologies [38] and with PROs [39]. Our study adds to a growing body of literature demonstrating how novel participatory methods can be used in conjunction with NPT to provide rich understandings to inform intervention development and implementation. This broadens the potential toolbox of methods for researches to draw from to perform NPT-guided data collection and analysis. For example, researchers focused on understanding interactions between stakeholders could employ NPT alongside forum theatre, as demonstrated by Duke and colleagues [40], whereas the mapping techniques used in the present study may be particularly appropriate for collaboratively planning delivery of new services.

A key finding in the study was the need for flexibility to enable recommendations to be enacted in different ways by different sites. Although differences in delivery can be considered a challenge to supporting implementation at scale, it is increasingly recognised in implementation that a ‘core and custom’ approach may be necessary. This aims to identify a central ‘core’ of an intervention but also a ‘soft periphery’ where local tailoring can occur [41, 42]. Embracing these elements early on in the process may therefore in fact help with achieving implementation at scale later on, through capturing examples of adaptation [43].

These findings underscore the need for implementation support which empathises with the users, their motivations, constraints, and concerns, and which explicitly expresses the value of the additional innovation work in meeting their needs. Allan and colleagues have described digital health data collection as successful if the data collected are “symbolically meaningful to each stakeholder’s role” (Page 9) [11]. This again emphasises the need for data to have value in achieving user goals, whether it is a nurse’s goal to understand their patient, or a patient’s goal to communicate changes that matter to them. Shaw and colleagues, in recommending a service design approach to digital health implementation, have described this as the requirement of a clear value proposition [36]. Our study has shown how a co-design and theory-driven approach can help to surface the potential value of ePRO data, and has shown how researchers need to more explicitly articulate this value for engagement to occur. Researchers therefore need greater temporal literacy regarding communicating the potential value, recognising that the anticipated end value of a new way of working must be expressed first, to encourage initial engagement. Coherence is a dynamic construct in practice, and it has been shown that collective action itself can help stakeholders develop understanding of value [44, 45]. Our finding that anticipated value was unclear at this preliminary stage of the project however indicates a need to communicate sufficient value to instigate engagement that can enable work to begin.

We make the following recommendations for future implementation co-design. Firstly, our observation revealed information that was not discussed in the workshops, for example the role of HCAs on the ward, and made clear that processes of work were actively negotiated in practice between professionals. Whilst not a limitation, we suggest future work may benefit from conducting workshops after an observation stage rather than before, as staff who are identified as important during the observation can then be included as participants in the co-design activities. Secondly, we recommend consideration of how to achieve staff and patient collective co-design, as we were logistically unable to achieve this. Adopting methods which do not require participants to be together at the same time may help (for example, co-design through suggestion boards which can be completed when convenient) but this may miss out on the potential richness of multi-stakeholder interaction [46]. Finally, the present study did specifically seek the perspective of sub-groups who are known to struggle with completion of ePROs (such as elderly patients and some ethnic minorities). Although patients who took part all had relevant lived experience, they were a self-selected sample. Further work should seek to engage with diverse groups, to understand the potentially unique barriers and needs that such groups may have.

## Conclusions

The study demonstrates how theory and methods can be combined to understand the socio-technical context in which different users operate, to sensitise recommendations not only for individual users but to understand how new digital ways of working can fit into relationships and organisational systems. The synthesis of methodology and conceptual framework enabled us to rapidly focus on recognised barriers and facilitators, to work collaboratively with sites to identify solutions to implementation challenges, and also to generate broader learning about the physical and intentional demands of introducing digital data collection into complex health services.

## Data Availability

The raw data are not available for sharing as we did not receive individual consent for this. Summary material used in qualitative analysis is available from the first author on request.
